# Potential Protein Biomarkers for Preeclampsia

**DOI:** 10.7759/cureus.8925

**Published:** 2020-06-30

**Authors:** Andong He, Yixuan Zhou, Yiling Wei, Ruiman Li

**Affiliations:** 1 Obstetrics and Gynecology, The First Affiliated Hospital of Jinan University, Guangzhou, CHN; 2 Obstetrics and Gynecology, The First Affiliate Hospital of Jinan University, Guangzhou, CHN

**Keywords:** preeclampsia, protein, proteomics, biomarkers, prediction

## Abstract

To date, the etiology of preeclampsia (PE) has not been clarified and the specific treatment is lacking; hence, early prediction and prevention are very important. Thus, a large number of biomarkers that may be associated with PE have been identified based on proteomics to provide a reference for the prediction of PE and for the understanding of the pathological mechanisms of this disease. This article briefly summarizes the application of proteomics in PE and the potential protein biomarkers to provide a reference for other researchers.

## Introduction and background

Pre-eclampsia (PE) is a complex pregnancy disease that affects 3% to 5% of pregnancy safety worldwide and is one of the important causes of maternal, fetal, and neonatal deaths [1]. To date, the pathogenesis of PE is not yet clear. Some researchers believed that the placenta plays an important role in its occurrence and development, as the patient's symptoms can be significantly relieved after the placenta is delivered [1-2]. Moreover, PE may be closely associated with maternal endothelial dysfunction and immune dysfunction [3]. Due to the lack of specific effective treatments for PE, its prediction and prevention are particularly important. A variety of prediction methods have been proposed, including biomarkers, maternal characteristics, Doppler ultrasound, or models composed of them [4]. If the result of prediction indicates high risk, oral aspirin may be recommended for prevention, although its effect needs further verification [5]. At present, researchers all over the world are committed to exploring biomarkers of PE, some of which are proteins, but no specific markers have been found yet. This article briefly summarizes the application of proteomics in the research for PE and famous protein biomarkers that may be related to PE to provide a reference for other researchers.

## Review

Application of proteomics

Omics technology has been widely used to screen biomarkers for various diseases. Therein, proteomics can be used to identify differentially expressed proteins between different samples. For understanding the functions and interactions of proteins, traditional techniques such as immunoblotting and enzyme-linked immunosorbent assay can provide a limited field of view, while mass spectrometry (MS)-based proteomics can be used to quantify thousands of proteins and to further identify their modification, location, and interaction. Currently, proteomics is generally based on liquid chromatography-mass spectrometry (LC-MS) or liquid chromatography-tandem mass spectrometry (LC-MS/MS) with high resolution, high accuracy, and high repeatability, which has been increasingly favored by researchers [6-7].

In recent years, proteomics has been applied to the study of PE, which can be used to identify biomarkers with the potential for prediction or diagnosis and can also provide a certain reference for understanding the pathological mechanisms of this disease [8]. For identifying disease-related biomarkers, maternal plasma or serum is generally used as a sample, and the placenta can also be used for studying biomarkers due to its vital role in some pregnancy diseases [9-11]. After identifying the differentially expressed protein, we can further study them by biological function analyses, including Gene Ontology (GO) analysis, Kyoto Encyclopedia of Genes and Genomes (KEGG) analysis, Protein-Protein Interaction (PPI) analysis, and others. At present, a large number of protein biomarkers that may be related to PE have been identified by the proteomics technology, including retinol-binding protein 4, gelsolin, clusterin, fibrinogen, fibronectin, and others. However, they all need to be further investigated [9-11].

Pro-angiogenic factor or anti-angiogenic factor

Normal placental angiogenesis is an essential foundation for forming great placental perfusion, establishing a suitable uterine environment, and ensuring the normal growth of the fetus. Pro-angiogenesis factors play a key role in this process [12]. It is currently believed that the imbalance of pro-angiogenic factors and anti-angiogenic factors is one of the pathogenesis of PE, as it can affect the remodeling of uterine spiral arteries and angiogenesis [13]. With reference to the existing literature, we briefly introduce several pro-angiogenic factors and anti-angiogenic factors that may be associated with PE, all of which are proteins.

Vascular Endothelial Growth Factor

Vascular endothelial growth factor (VEGF) is a kind of protein composed of macrophages, T cells, and cytotrophoblasts [12]. It has multiple subtypes, such as VEGF-A, VEGF-B, VEGF-C, VEGF-D, VEGF-E, VEGF-F, and placental growth factor (PlGF), which can specifically bind to VEGF receptors, including VEGFR-1, VEGFR-2, and VEGFR-3 [13]. VEGF can promote angiogenesis, such as tumor angiogenesis; hence, an anti-tumor therapy had been developed based on anti-VEGF [14]. At the same time, VEGF can increase vascular permeability and vasodilation through nitric oxide, thereby reducing vascular tone and blood pressure [15]. VEGF may also play an important role in the development of PE; Levine *et al*. found that the level of VEGF in the blood of patients during PE and within 5 weeks before PE was reduced, but it cannot predict whether it will happen later [16]. Kurtoglu *et al*. found that the serum VEGF in patients with severe PE was significantly higher than that in patients with mild PE and normal pregnant women and believed that VEGF may be an important indicator for predicting the severity of PE, but there is still insufficient evidence [17]. The mechanism and level of VEGF in PE need further study.

*Soluble Fms-like Tyrosine Kinase 1 (sFlt1) and PlGF* 

sFlt1, an anti-angiogenic factor, is a member of the type III receptor tyrosine kinase family, while PlGF is a pro-angiogenic factor. They can be released into the blood of pregnant women from the placenta, therein sFlt1 is an antagonist of PlGF and VEGF, which can cause endothelial dysfunction by blocking the receptor-binding domain of them [18]. Besides, Yonekura *et al*. found that placental sFlt1 is associated with damage to placental syncytiotrophoblasts and complement activation in PE [19]. Thus, sFlt1 may be an essential factor in the pathogenesis of PE. Studies have shown that sFlt1 increases in placenta or blood in patients with PE while PlGF decreases, and the increase of sFlt1 may be related to the decrease of VEGF and PlGF in the blood [15-16]. For predicting the onset of PE, sFlt1 and PlGF have shown high sensitivity from the second trimester of pregnancy [20]. It was further found that the increased sFlt1:PlGF ratio may have a certain value for predicting PE [21]. Zeisler *et al*. conducted a multi-center, prospective, and observational clinical study about the suspected PE pregnant women with singleton pregnancies between 24-37 weeks gestation [22]. In the first cohort, the sFlt1:PlGF ratio of 38 was found to be a boundary between the occurrence of PE and the absence of PE within the next week through the study of 500 pregnant women; and in the second cohort, a study of 550 pregnant women indicated that the negative predictive value at an sFlt1:PlGF ratio of 38 or lower was 99.3% (95% confidence interval, 97.9-99.9), that is, the probability of such pregnant women developing PE in the following week is very low, while the positive predictive value of sFlt1: PlGF ratio above 38 for PE occurrence within the next 4 weeks was only 36.7 (95% confidence interval, 28.4-45.7). However, the study also has some limitations, for example, the objects were only the women with singleton pregnancies, and their gestational ages were limited to a fixed range. It is worth noting that some clinical studies suggest that sFlt1 and PlGF have limited ability to predict PE [23]. In a word, due to the potential role of sFlt1 and PlGF in the pathogenesis of PE and their predictive ability, they have become the star members in PE biomarkers, but more clinical trials, especially randomized trials, still need to be evaluated in the future to assess their values.

Soluble Endoglin

Endoglin is a membrane-bound protein that promotes nitric oxide production by endothelial cells and inhibits apoptosis [24]. Soluble endoglin (sENG) is a soluble isomer of endoglin, which plays an anti-angiogenic role by inhibiting the binding of transforming growth factor-β1 (TGF-β1) to its receptor on endothelial cells. It can affect the permeability of blood vessels in vivo to cause high blood pressure, and can also inhibit capillary angiogenesis in vitro [25]. Studies have shown that serum sENG can increase in the last two months of pregnancy in normal pregnancy, but faster in PE and peak during the onset of the disease, and it may be associated with the severity of the disease. Moreover, it may play a synergistic role with sFlt1 in the pathogenesis of PE [18,25-26]. Therefore, some researchers believed that increased sENG in the blood and increased sFlt1:PlGF ratio can predict the occurrence of PE, but more clinical data are still needed to verify it [18].

Pregnancy-associated plasma protein-A

Pregnancy-associated plasma protein-A (PAPP-A) is a glycoprotein, mainly produced in placental trophoblast cells, which can reflect the degree of placental ischemia or hypoxia. PAPP-A can regulate the activity of insulin-like growth factors, thereby affecting the infiltration of placental trophoblast cells; hence, it is very important for normal pregnancy [27]. PAPP-A can be secreted from the placenta into the blood, which is often used for aneuploidy screening during early pregnancy, and it means an increased risk of trisomy when its level is low [28]. At the same time, low serum levels of PAPP-A during the early pregnancy may also be associated with PE, and its ability to predict PE during the second trimester is poor, while the serum PAPP-A in patients with PE during the late pregnancy can be high but it cannot be used as a predictor of the severity of PE [29-31]. Furthermore, some researchers believed that PAPP-A needs to be combined with Doppler ultrasound to predict PE to obtain higher sensitivity [20]. However, these conclusions are subject to further verification.

Placental protein 13

Placental protein 13 (PP13) is also called Galectin 13 (Gal-13). Galectin is a type of carbohydrate-binding protein, whose family is related to inflammation, immune response, and apoptosis. Some galectins play a key role in the regulation of reproductive system functions. PP13 has a high affinity for sugar residues, such as the sugar residues of AB and B antigens in the ABO blood group. It can play an essential role in pregnancy by interacting with glycoproteins and glycolipids, including promoting uterine arteriovenous dilatation during pregnancy and maintaining the stability of maternal vascular structure [32]. Some studies have found that the serum PP13 in patients with PE is low during early pregnancy, which can be considered as a biomarker to predict PE [33-35]. Through a case-control study, Shimizu *et al*. found that the mRNA level of PP13 in blood cells of PE patients with or without symptoms was significantly lower than that of normal pregnant women [36]. The decreased expression of PP13 in the placenta may also be related to PE [37]. In addition, Nicolaides *et al*. found that patients with PE who need to be delivered before 34 weeks of gestation can be screened by a joint evaluation of serum PP13 levels during the early pregnancy and uterine artery Doppler examination [34]. Giguère *et al*. also found that the combination of biomarkers and uterine artery Doppler examination can improve the predictive effect of PE by a systematic review, in which PP13 can be combined with PPAP-A, integrin-like metalloproteinase 12, activin, inhibin, uterus arterial Doppler examination or others [38]. PP13 has a certain value for predicting PE, but more data are still needed for evaluation.

Heat shock protein

Heat shock protein (HSP), a highly conserved protein, is widely present in organisms or cells, which plays a key role in the formation of protein complexes, cell cycle regulation, and immune regulation [39]. As for the relationship between HSP and PE, the current research is more about HSP70. HSP70 participates in various physiological processes such as protein folding, and it can protect cells from apoptosis. HSP70 expression can be mediated by placental ischemia, oxidative stress, and maternal inflammatory response. Peracoli *et al*. found that HSP70 may be related to a variety of pro-inflammatory cytokines, including tumor necrosis factor-α, interleukin-1β, interleukin-12, and others [40]. Overexpressed HSP70 may play a role in the pathogenesis of salt-induced hypertension [41]. Studies had found that serum HSP70 in PE patients was significantly higher, but Livingston *et al*. [found that the serum HSP70 in patients with severe PE was not higher than that in normal pregnant women [42-44]. Based on this disagreement, Saghafi *et al.* conducted a meta-analysis and found that the serum HSP70 in patients with PE was significantly higher than that in normal pregnant women, although only seven studies were included [45]. Moreover, Hromadnikova *et al.* found that the mRNA level of HSP70 in patients with PE is high, but it has nothing to do with the severity of the disease [46] . Thus, HSP70 may be considered for the diagnosis of PE, but the current relevant evidence mainly comes from case-control studies, and there is still a lack of large-scale prospective studies.

Fetal hemoglobin

Fetal hemoglobin (HbF) can induce oxidative stress that is considered to be closely related to PE. Oxidative stress can be found in the placenta and blood of patients with PE. Thus, in recent years, researchers have explored the relationship between HbF and PE [47]. Through prospective study, Centlow *et al*. found that the production of HbF in the placenta of patients with PE was significantly increased and could be released into the vascular cavity of the placenta, which was considered to be related to endothelial damage and inflammation [48]. Therefore, HbF may play a role in the progression of PE by damaging the placenta, kidney, and other tissues, although its specific mechanism needs to be further studied. Notably, α1-microglobulin (A1M) is an antioxidant, and its expression in the placenta of PE had been found to be increased, which may be regulated by the feedback of placental oxidative stress. A1M has a certain offset effect on the tissue damage caused by HbF. Therefore, A1M may be a potential direction for the study of the treatment of PE [47]. HbF can be released into the blood of patients with PE because oxidative stress can damage the blood-placental barrier, and the serum HbF in patients with PE was found to be elevated during the early pregnancy, so HbF has a certain value in the prediction of PE [49]. Recently, Bellos *et al*. indicated that the elevated HbF and A1M were associated with PE by a meta-analysis, but more large-scale clinical studies are still needed to explore their value in the prediction of PE [50].

## Conclusions

Strengthening the study on the etiology, prediction, prevention, and treatment of PE is an important task for researchers all over the world. At present, proteomics and other technologies are widely used in the study of PE. Many protein biomarkers that may be related to PE have been identified. Among them, VEGF, sFlt1, PlGF, sENG, PAPP-A, PP13, HSP70, HbF, and other proteins have shown a certain value in the prediction or diagnosis of PE and help understand the pathogenesis of PE. However, there is currently no specific protein marker for PE. It is still necessary for us to explore it through more clinical trials, experiments, and omics analysis.
